# Comparison of viral load between saliva and nasopharyngeal swabs for SARS-CoV2: the role of days of symptoms onset on diagnosis

**DOI:** 10.1590/0074-02760210018

**Published:** 2021-04-16

**Authors:** Alberto Fernando Oliveira Justo, Mariana Sardinha Bueno, Gabriela Rodrigues Barbosa, Ana Helena Perosa, Joseane MA Carvalho, Nancy Bellei

**Affiliations:** 1 Universidade Federal de São Paulo, Departamento de Medicina, Laboratório de Virologia Clínica, São Paulo, SP, Brasil; 2 Hospital São Paulo, Laboratório Central, São Paulo, SP, Brasil

**Keywords:** COVID-19, viral load, RT-PCR

## Abstract

**BACKGROUND:**

Coronavirus disease 2019 (COVID-19) is highly infectious causing millions of deaths worldwide. Nasopharyngeal swabs are the primary sample of choice for the diagnosis of severe acute respiratory syndrome coronavirus 2 (SARS-CoV-2), thus, to decrease the exposure to potentially infected samples through the collection is a key point to reduce the risk of infection in healthcare workers.

**OBJECTIVES:**

This study aimed to evaluate the sensitivity and viral load of saliva specimens by days of symptoms onset comparing to nasopharyngeal swabs in subjects with mild symptoms.

**METHODS:**

Saliva and nasopharyngeal swabs samples were collected from São Paulo Hospital workers presenting mild symptoms, such as fever, cough, sore throat, rhinorrhea, myalgia, headaches, anosmia, ageusia, and fatigue. To understand the positivity and viral load, reverse transcription-polymerase chain reaction (RT-PCR) was performed.

**FINDINGS:**

Saliva specimens presented a sensitivity of 98.6% compared to nasopharyngeal swabs. Overall, saliva showed lower viral load compared to nasopharyngeal swabs, regarding days of symptoms onset on diagnosis, the first four days had significant changes in viral load and no significant difference was reported in the days five to nine.

**MAIN CONCLUSIONS:**

Although RT-PCR of saliva has presented a lower viral load compared to nasopharyngeal swabs, saliva specimens are a potential and reliable candidate for COVID-19 diagnosis through RT-PCR.

Coronavirus disease 2019 (COVID-19) has emerged as the main public health issue of the first quarter of the century. This reputation is related to the highly infectious characteristics of this disease, and its impact in several systems, such as vascular, coagulation, and respiratory. According to the most recent report (16 March 2021) from the World Health Organization (WHO), 119 million people were infected and 2.6 million lost their lives worldwide, from those, Brazil counts for 11 million infected people and 278 thousand deaths.^1^

The rate of symptomatic and asymptomatic subjects infected by COVID-19 has not yet been well established, however, it is estimated that asymptomatic subjects can range from 20 to 80%, mild symptomatic 10-30%, and 5-20% moderate and severe symptomatic. It is important to note that lethality of this disease can be approximately 2-5%, mainly in subjects who present risk factors, such as advanced age, obesity, diabetes mellitus, hypertension, cardiovascular and cerebrovascular disease.^2^ ^,^ ^3^ ^,^ ^4^ The transmission can occur via contaminated nasal/oral respiratory droplets and surfaces.^5^

Molecular biology approaches are the standard methods to detect coronavirus. The most reliable and widely used COVID-19 diagnostic test is the reverse-transcriptase polymerase chain reaction (RT-PCR).^6^ Although, the biological specimens to be analysed by RT-PCR can differ depending on the severity of the disease, day of the onset of the symptoms, and whether the patient is hospitalised or not. For RT-PCR diagnosis, the most utilised samples are naso and/or oropharyngeal swabs, feces sputum, and bronchoalveolar lavage.^6^ ^,^ ^7^ However, the collection of those samples from a potentially infected subject requires healthcare workers that are exposed to the infection because of direct contact to the patients.

Regarding the COVID-19 incidence, the healthcare worker has approximately 10-fold times higher incidence compared to the general community (3.96 vs 0.33%).^8^ In our previous study,^9^ we showed that nursing technicians, nurses, and physicians are the most infected among the healthcare workers, respectively. Although it is unclear the reason that nursing technicians are the most affected, it is hypothesised that these healthcare workers are more exposed by physical contact with potentially infected patients, including in the collection of samples.

In the sense of the high transmission of COVID-19, it is important to reach more options of diagnosis, since the most utilised by now can be infectious to healthcare workers and uncomfortable to the subjects. In this context, we aimed to evaluate the sensitivity of saliva specimens comparing to nasopharyngeal swabs from mild symptomatic subjects. We also aim to investigate whether the day of the symptoms onset play a role on viral load.

## SUBJECTS AND METHODS

*Sample collection* - The study was conducted at the Laboratory of Clinical Virology, São Paulo Hospital, Brazil. Nasopharyngeal swabs were collected from healthcare workers presenting mild symptoms, such as fever, cough, sore throat, rhinorrhea, myalgia, headaches, anosmia, ageusia, and fatigue. At the same time, the subjects were asked to self-collect 2 mL of their saliva in a sterile tube, avoiding mucous secretions from the oropharynx and sputum. This study was conducted in compliance with institutional guidelines, approved by the Ethics Committee of São Paulo Federal University (CEP/UNIFESP n. 4.013.602) and all individuals signed written informed consent forms.

*RT-PCR* - RNA was isolated from 300 µL of the subject’s nasopharyngeal swabs and saliva using the Quick-RNA Viral Kit (Zymo Research, Irvine, CA), according to the manufacturer protocol. After extraction, the RNA was used immediately, and the remained RNA was stored at -80**º**C. The samples were tested for severe acute respiratory syndrome coronavirus 2 (SARS-CoV-2) using a multiplex RT-PCR commercial kit (GeneFinder, OSANG, South Korea) which targets the genes RdRp (RNA-dependent RNA Polymerase), E (envelope), and N (nucleocapsid) for SARS-CoV-2. The reaction was carried out as described in Table I.

**TABLE I:** Conditions for reverse transcription-polymerase chain reaction (RT-PCR)

Cycles		Temperature (ºC)	Time
Reverse transcription		50	20 min
Enzyme inactivation		95	5 min
X 45 cycles	Denaturation	95	15 s
	Annealing and extension	58	60 s

For interpretation of results, the cycle threshold (Ct) values were used as an indicator of the viral load of the SARS-CoV-2 RNA. The Ct values generated by RT-PCR provide inversely proportional measurements of viral load. A positive result was considered with a Ct value to lower than 40, for at least two SARS-CoV-2 genes (RdRp, E or and N).

*Equation for variation of Ct* - For the analysis of the difference between the Cts from the specimens of saliva and NP swabs from the same subject an equation was calculated, as described below:

*Statistical analysis* - The program GraphPad Prism version 6 was used for all statistical analysis. Statistical analysis consisted of Student’s t-test for comparations, with a p < 0.05 being considered statistically significant. Results are expressed as mean ± standard deviation (SD).

## RESULTS

From late October to early December, 76 nasopharyngeal swabs and saliva from the same subject were collected and the RT-PCR was performed in the Laboratory of Clinical Virology from São Paulo Hospital. The mean age of the subjects was 34.9 years (ranging from 19 to 70); 23 individuals (30.2%) were men and 53 (69.8%) were women.

**Fig. 1:**
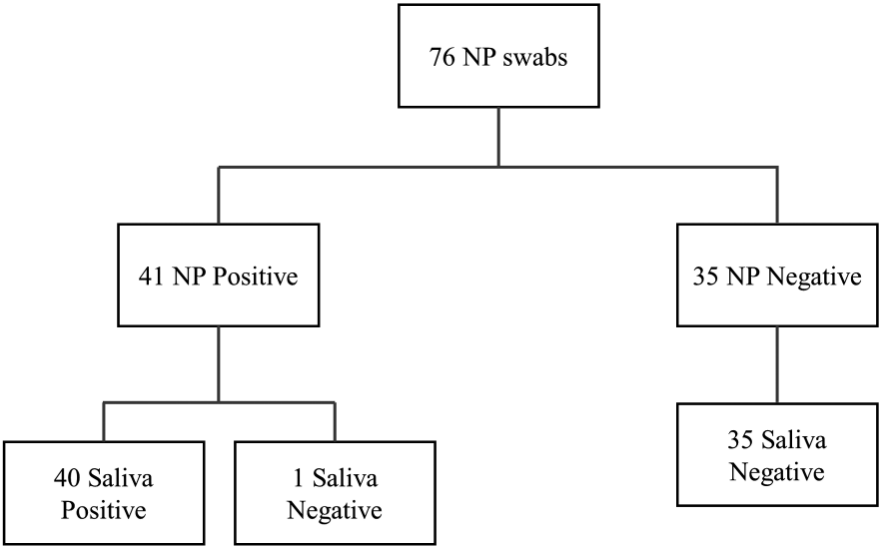
entrance scheme of specimens results of reverse transcription-polymerase chain reaction (RT-PCR) for severe acute respiratory syndrome coronavirus 2 (SARS-CoV-2).

From all nasopharyngeal samples, 54% tested positive and 46% negative. The sensitivity was 98.6% comparing to saliva RT-PCR, as shown in Fig. 1. In all negative nasopharyngeal swabs samples, saliva RT-PCR had 100% of the sensitivity of the results, although, when compared to the positive results, the sensitivity was 97.56%.

**Fig. 2:**
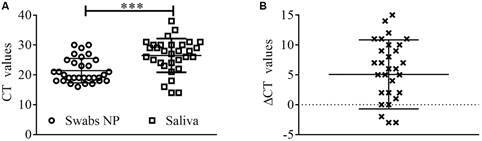
comparison between nasopharyngeal swabs and saliva. (A) Cycle threshold (Ct) values from the nasopharyngeal swabs and saliva. (B) Difference between Ct of saliva and nasopharyngeal. Values represent the mean ± standard deviation (SD); ***p < 0.001; NP: nasopharyngeal.

In order to understand the viral load from the clinical specimens, nasopharyngeal swabs, and saliva samples the RT-PCR Cts were analysed. Interestingly, RT-PCR showed that nasopharyngeal swabs presented lower Ct 21.42 **±** 4.10 (Fig. 2A; p < 0.001) comparing to saliva Ct 26.48 **±** 5.65. To analyse the difference of those, we calculated the ΔCt, as previously described. Nasopharyngeal swabs showed lower Ct comparing to saliva (mean 5.06 **±** 5.77) **(** Fig. 2B) although, five out of 40 saliva samples presented higher Cts compared to nasopharyngeal swabs, two were the same in both samples, and 33 out of 40 had higher Cts in nasopharyngeal swabs.

Since the overall nasopharyngeal swabs had a higher viral load compared to saliva, we aimed to understand whether the onset of the symptoms had any effect on Cts from nasopharyngeal swabs and saliva (Table II). Nasopharyngeal swabs had lower CTs in all days of symptoms onset compared to saliva, although, a significant difference was seen only on days one-two (p = 0.002) and three-four (p = 0.04).

**TABLE II:** Days of symptoms onset until sample collection and cycle threshold (Ct) of subjects (n = 40)

Symptoms onset (days)	Patients (n)	Ct NP swab (mean ± SD)	Ct saliva Ct (mean ± SD)	p value
One - two	10	21.20 ± 4.29	27.40 ± 3.47	**0.002
Three - four	18	23.50 ± 6.29	28.00 ± 6.79	*0.04
Five - six	6	20.00 ± 2.00	24.20 ± 6.61	0.21
Seven - nine	6	29.80 ± 7.79	34.40 ± 4.98	0.29

*: p < 0.05; **: p < 0.01; NP: nasopharyngeal; SD: standard deviation.

## DISCUSSION

The results of this study highlight the reliability of saliva specimens in symptomatic patients, as a valuable sample for COVID-19 diagnosis. Saliva RT-PCR showed a sensitivity of 98.6% comparing to nasopharyngeal swabs RT-PCR, which is the reference method. Although, it is important to note that the viral load was higher in nasopharyngeal swabs comparing to saliva in the first four days of symptoms onset and no significant difference in Ct of the days five to nine was observed.

Choosing the most appropriate clinical specimens in COVID-19 diagnosis remains challenging. A previous report^10^ showed that specimens collected from 204 patients diagnosed with COVID-19 had the highest RT-PCR positivity rates in bronchoalveolar lavage fluid (93%) followed by sputum (72%), nasal swabs (63%), pharyngeal swabs (32%), feces (29%), blood (1%) and none positive in urine specimens. Apart from asymptomatic subjects, those with mild symptoms constitute the majority of the COVID-19 cases, making the collection of nasopharyngeal or oropharyngeal for RT-PCR the most reasonable. However, the collection of these samples can be invasive and painful for the individual; thus, it is required specialised healthcare workers, which manipulate potential infectious samples, whilst saliva can be collected by the subjects themselves, following the healthcare professional instruction.^11^

Despite the collection of clinical specimens, it is important to highlight the costs. For example, apart from the RT-PCR per se, the collection of naso/oropharyngeal specimens costs approximately R$ 2,15 per sample including a swab, tube, and viral transport medium.^12^ On the other hand, for saliva, the proper collection does not require swab and viral transport medium, reducing the cost by less than half.

In this study, the nasopharyngeal swab and saliva samples were collected from São Paulo Hospital health workers who presented only mild symptoms. The saliva RT-PCR showed a sensitivity rate of 98.6% compared to nasopharyngeal swabs, however, the Ct was higher, which indicates that the viral load is lower in saliva. A previous study^13^ with hospitalised patients showed that RT-PCR from naso/oropharyngeal swabs and saliva collected on the third day of hospitalisation had a sensitivity of 70% (21 out of 30). However, it is important to address some factors that may play a role in the difference of sensitivity of the tests: (i) severity of symptoms, (ii) days of symptoms onset in hospitalised patients, and (iii) the number of subjects included in the study.

Similar to our study, a previous study^14^ with mild symptomatic subjects showed that the sensitivity of nasopharyngeal swab and saliva RT-PCR was 83.3% (15 out of 18) and the Ct value was significantly higher in saliva samples. In our study, independent of days of symptoms onset, saliva had a mean of five Cts higher compared to nasopharyngeal swab, which suggests that the viral load is lower in saliva. In order to understand the potential role of days of symptoms onset, we analysed by days; from day 1 to day 9, consistently, the nasopharyngeal Ct was lower for the saliva samples. However, the first four days presented a significant difference between Cts, and from day five to nine, no significant change was seen.

Rapid test using saliva is emerging as a potential test for COVID-19 diagnosis and it is getting more popular in Brazil and worldwide. Previous studies^15^ ^,^ ^16^ have shown that the Ct from saliva is an important factor for the successful result of the tests and when the Ct is up to 30, it is related to a more reliable test, which indicates higher viral load in the specimen. Our study suggests that the first six days of symptoms are the most appropriate for the saliva rapid test.

It is widely known that healthcare workers are one of the most exposed groups, hence, more infected by SARS-CoV-2. In earlier cases from Wuhan, China, approximately 29% of the patients infected by COVID-19 were healthcare workers and it was assumed that the infection occurred during working time.^17^ The collection of specimens such as naso/oropharyngeal swabs and bronchoalveolar lavage fluid can increase the odds of infection from patient to healthcare worker by close contact with biological samples. Saliva is a potential specimen for RT-PCR as well as rapid tests.^13^ Besides the cost, the self-collection of saliva is one of the major advantages of this specimen since the healthcare worker has no direct contact during collection.

Collectively, we present here an observational study report which analyses the RT-PCR sensitivity and viral load from saliva specimen comparing to nasopharyngeal swab from 76 subjects with mild symptoms subjects. The sensitivity of saliva was 98.6% when compared to nasopharyngeal swabs. Although, the viral load evaluated by the Ct, was significantly lower in saliva samples, particularly in the first 4 days of symptoms onset. Our findings indicate that saliva is as efficient as a nasopharyngeal swab for RT-PCR, and it is advantageous for lower cost and decrease of the exposure of healthcare workers collecting samples from potentially infected subjects.

## References

[1] WHO (2021). COVID-19 epidemiological update [Internet]. World Health Organization.

[2] Dantés HG, Manrique-Saide P, Vazquez-Prokopec G, Morales FC, Siqueira JB, Pimenta F (2020). Prevention and control of Aedes transmitted infections in the post-pandemic scenario of COVID-19 challenges and opportunities for the region of the Americas. Mem Inst Oswaldo Cruz.

[3] Oran DP, Topol EJ (2021). The proportion of SARS-CoV-2 infections that are asymptomatic. Ann Intern Med.

[4] Gandhi RT, Lynch JB, del Rio C (2020). Mild or moderate Covid-19. N Engl J Med.

[5] Senger MR, Evangelista TCS, Dantas RF, Santana MVS, Gonçalves LCS, de Souza Neto LR (2020). COVID-19 molecular targets, drug repurposing and new avenues for drug discovery. Mem Inst Oswaldo Cruz.

[6] Sood S, Aggarwal V, Aggarwal D, Upadhyay SK, Sak K, Tuli HS (2020). COVID-19 pandemic from molecular biology, pathogenesis, detection, and treatment to global societal impact. Curr Pharmacol Reports.

[7] Moreira LVL, de Souza Luna LK, Barbosa GR, Conte DD, Carvalho JMA, Bellei N (2020). Test on stool samples improves the diagnosis of hospitalized patients: detection of SARS-CoV-2 genomic and subgenomic RNA. J Infect.

[8] Nguyen LH, Drew DA, Graham MS, Joshi AD, Guo C-G, Ma W (2020). Risk of COVID-19 among front-line health-care workers and the general community a prospective cohort study. Lancet Public Health.

[9] Faíco-Filho KS, Carvalho JMA, Conte DD, de Souza Luna LK.Bellei N (2020). COVID-19 in health care workers in a university hospital during the quarantine in São Paulo city. Braz J Infect Dis.

[10] Wang D, Hu B, Hu C, Zhu F, Liu X, Zhang J (2020). Detection of SARS-CoV-2 in different types of clinical specimens. J Am Med Assoc.

[11] Bastos ML, Perlman-Arrow S, Menzies D, Campbell JR (2021). The sensitivity and costs of testing for SARS-CoV-2 infection with saliva versus nasopharyngeal swabs : a systematic review and meta-analysis. Ann Intern Med.

[12] Gil A, Neves M, Rios-Neto ELG, Carvalho F, Dickman R (2020). Uma alternativa para o aumento da escala da testagem para a Covid-19.

[13] Güçlü E, Koroglu M, Yürümez Y, Toptan H, Kose E, Güneysu F (2020). Comparison of saliva and oro-nasopharyngeal swab sample in the molecular diagnosis of COVID-19. Rev Assoc Med Bras.

[14] Skolimowska K, Rayment M, Jones R, Madona P, Moore LSP, Randell P (2020). Non-invasive saliva specimens for the diagnosis of COVID-19 caution in mild outpatient cohorts with low prevalence. Clin Microbiol Infect.

[15] Agulló V, Fernández-González M, Ortiz de la Tabla V.Gonzalo-Jiménez N.García JA.Masiá M (2020). Evaluation of the rapid antigen test Panbio COVID-19 in saliva and nasal swabs in a population-based point-of-care study. J Infect.

[16] SoRelle JA, Mahimainathan L, McGormick-Baw C, Cavuoti D, Lee F, Thomas A (2020). Saliva for use with a point of care assay for the rapid diagnosis of COVID-19. Clin Chim Acta J.

[17] Bielicki JA, Duval X, Gobat N, Goossens H, Koopmans M, Tacconelli E (2020). Monitoring approaches for health-care workers during the COVID-19 pandemic. Lancet Infect Dis.

